# Potential Effects of Lung Function Reduction on Health-Related Quality of Life

**DOI:** 10.3390/ijerph16020260

**Published:** 2019-01-17

**Authors:** Yuhan Wen, Dongming Wang, Min Zhou, Yun Zhou, Yanjun Guo, Weihong Chen

**Affiliations:** 1 Department of Occupational & Environmental Health, School of Public Health, Tongji Medical College, Huazhong University of Science and Technology, Wuhan 430030, China; wenyuhan@hotmail.com (Y.W.); wangdongming2008@126.com (D.W.); mzhou2016@hust.edu.cn (M.Z.); sarayunzhou@gmail.com (Y.Z.); hustghkan@126.com (Y.G.); 2 Key Laboratory of Environment and Health, Ministry of Education & Ministry of Environmental Protection, and State Key Laboratory of Environmental Health (Incubating), School of Public Health, Tongji Medical College, Huazhong University of Science and Technology, Wuhan 430030, China

**Keywords:** forced vital capacity, forced expiratory volume, lung disease, health-related quality of life

## Abstract

Health-related quality of life (HRQOL) was reported to reflect overall quality of life and individual perceptions related to health. Decreased lung function is associated with reduced ventilation and oxygen intake and reported to affect body functions. However, the effect of lung function reduction on HRQOL is still unclear. A total of 8398 retired workers from Dongfeng-Tongji Cohort Study were included in this cross section study. Lung function was measured using an electronic spirometer. HRQOL was evaluated through a questionnaire designed according to the WHOQOL-BREF. The mean of the HRQOL scores of its four domains (physical health, psychological state, social relationships and environment) is the total HRQOL score. A general linear model was used to analyse the association between lung function and HRQOL. In the continuous analysis by the general linear model, FVC was associated with the total HRQOL, physical health domain and social relationships domain scores. In the categorical analysis, there was a linear trend between FVC and the total HRQOL and physical health scores. We also found a similar relationship between FEV_1_ and HRQOL scores. Further analysis suggested that elevated lung function could improve the scores of pain and discomfort facet and independence facet of physical health domain. The lung function was significantly positively associated with HRQOL in middle-aged and older Chinese.

## 1. Introduction

Health-related quality of life (HRQOL) refers to the dimension of health impact on the quality of life. According to the definition suggested by the World Health Organization Quality of Life Group, HRQOL could reflect the perceptions of individuals in different cultures and value systems with respect to their goals, expectations, standards, and living conditions related to their concerns [1]. In recent years, HRQOL has caught more and more attention and became a hot topic because HRQOL is used not only to assess the impact of physical condition on life, but also to evaluate possible effects of various factors such as medical service systems, living habits and social welfare on daily life [2,3]. Normally, HRQOL is evaluated by a questionnaire designed according to the World Health Organization Quality of Life-BREF. This questionnaire yields a multi-dimensional profile of score across four domains which include physical health, psychological state, social relationships and environment. In physical health domain, three facets of pain and discomfort, energy and fatigue and independence could be evaluated through the questionnaire [1]. In current study, we wanted to understand the potential effects of lung function reduction on HRQOL.

As the Institute for Health Metrics and Evaluation (IHME) showed in 2017, chronic respiratory diseases (CRDs) were the fourth leading global cause of death, and the DALYs of CRDs was 1470.03 per 100,000. Lung function is an important parameter for complete evaluation of respiratory system function, including airway ventilation capacity and physical condition. Decline in lung function has been used in the clinic diagnosis of airway dysfunction or diseases including CRDs. Decreased lung function is associated with reduced ventilation and oxygen intake, especially during intense activity. In addition, reduced lung function could also affect other body functions such as sleep [4]. All of these might have an adverse effect on HRQOL. Recently, accumulating evidences have found significant correlations between forced expiratory volume in one second (FEV_1_) and HRQOL in patients with COPD [5]. Berry et al. also suggested that the relationship between FEV_1_ and HRQOL scores was linear and statistically significant in patients with chronic respiratory diseases [6]. Some other studies have shown that the HRQOL scores in patients with lung diseases such as COPD were lower than those of healthy participants [7,8]. The HRQOL scores gradually decreased with the increasing severity of COPD [9,10,11,12]. Furthermore, the negative effects of lung function decline or COPD on HRQOL included physical domain and psychosocial domain. With respect to the different domains of HRQOL, the results of another study were inconsistent. A 12-year longitudinal study suggested that it was not the physical domain but the psychosocial domain that decreased the most with the declining lung function [13]. Kim et al. suggested that the total HRQOL and all HRQOL domains were associated with disease severity in a large population of COPD patients [14]. Garcia-Gordillo et al. found that in COPD patients the reduction was stronger on the physical than on the mental component of HRQOL [15]. However, the potential effects of lung function level on HRQOL among general population are still unclear.

Therefore, we developed this study using baseline data of 12,344 retired workers in Dongfeng-Tongji Cohort Study. The objectives of this study were to explore the relationship between lung function reduction and HRQOL in middle-age and older people, and identify which domain of HRQOL was impacted by lung function level. The hypothesis was that lung function was significantly positively associated with HRQOL.

## 2. Materials and Methods

### 2.1. Study Subjects

The study was embedded in the Dongfeng-Tongji Cohort Study, which has been reported elsewhere [16]. The cohort study was established in 2008 and was followed up every five years. Finally, a total of 27,009 retired employees completed baseline questionnaires and medical examinations from September 2008 to June 2010. Five years later, a total of 12,344 retired workers who finished lung function test and HRQOL assessment in 2013 were recruited in this cross section study. In the present study, participants with a history of cancer (*n* = 726), coronary heart disease (*n* = 1824), myocardial infarction (*n* = 377), stroke (*n* = 449), hepatitis (*n* = 431), nephritis (*n* = 414), head trauma (*n* = 523) or missing data (*n* = 143) were excluded (there might be someone who had more than one disease). Finally 8398 participants were included in the analyses. The sample size of our study was larger than the minimum sample size (*n* = 1621), which was calculated by the sample size calculation formula.

### 2.2. Lung Function

Lung function was measured using an electronic spirometer (Chest graph HI-101, CHEST Ltd., Tokyo, Japan). The electronic spirometer device was calibrated before each test, according to the manufacturer’s instruction. The volume precision of the device is ±3% or ±50 mL. The participants were advised not to smoke or eat for at least 1 h before the test. All participants remained in a sitting position, wore a nose clip, and breathed through the mouth during the test. Each participant completed three tests, of which the highest value was used in the analysis. We used forced vital capacity (FVC), FEV_1_, percent of predicted FVC (%pred FVC) and microairway indexes such as maximal mid-expiratory flow curve (MMF), peak expiratory flow (PEF), forced expiratory flow after 25% of vital capacity has been expelled (FEF_25_), forced expiratory flow after 50% of vital capacity has been expelled (FEF_50_), and forced expiratory flow after 75% of vital capacity has been expelled (FEF_75_) to assess lung function. According to the results of lung function and self-reported respiratory disease (diagnosed by doctor or take drugs), the participants were divided into two groups. The poor lung function group included individuals with FVC < 80% or self-reported asthma, bronchitis, emphysema, COPD or interstitial lung disease (ILD). Others were divided into normal lung function group [17].

### 2.3. Assessment of HRQOL Scale

The HRQOL scale was estimated through face to face interviews using the questionnaire designed according to the World Health Organization Quality of Life-BREF. Internal reliability of the questionnaire was demonstrated using Cronbach’s α coefficients (α = 0.81). It includes four domains of HRQOL: physical health, psychological state, social relationships and environment. Domain scores were calculated by the mean of all items included within the domain. We then converted scores to percentage system and the possible range for the converted score was 0 to 100. The converted score was calculated as follows:

Converted score = (original score − 4) × 100/16,(1)

The total HRQOL score is the mean of four domains scores. Higher scores indicate better HRQOL. In addition, physical health domain includes three facets: pain and discomfort, energy and fatigue and independence. The facet score was calculated by the mean of all items included within the facet [1,2].

### 2.4. Covariates

The data on demographics (birth year, and gender), medical history, and lifestyle (smoking, alcohol drinking, and physical exercise) were collected through a semi-structured questionnaire by face to face interviews with trained interviewers. After that, all participants took lung function tests and conventional physical examinations. Current smoking was defined as having at least 1 cigarette per day for more than 6 months, and those who had ever smoked and have quit smoking for more than 6 months defined as quit smokers. Current drinking was defined as the consumption of alcohol at least once a week for more than 6 months. Physical activity was defined as regularly exercised for more than 20 min per day and more than 3 times per week over the last six months. Educational level was classified into three levels: less than high school (<9 years), higher school (9–12 years) and completed a university degree or above (>12 years). Marriage status was classified as married and single. Body mass index (BMI) was calculated as weight divided by height squared (kg/m^2^). Chronic diseases history diagnosed by a physician was reported by the participants themselves. And comorbidities included gallstone, rhinitis, hypertension, hyperlipemia or diabetes.

### 2.5. Ethical Approval

All subjects gave their informed consent for inclusion before they participated in the study. The study was conducted in accordance with the Declaration of Helsinki, and the protocol was approved by the Ethics Committee of Dongfeng General Hospital, Dongfeng Motor Corporation, and the Medical Ethics Committee of the School of Public Health, Tongji Medical College, Huazhong University of Science and Technology (Identification code: (2010–16); Wuhan, China, May 2008).

### 2.6. Statistical Analysis

Quantitative variables were expressed as mean ± SD, and categorical variables were expressed as number and percentages. Student’s *t*-test and Kruskal-Wallis test were used to analyze differences in the normal distribution variables and abnormal distribution variables between two groups, respectively. Chi-square test was used to analyze categorical variables in two groups.

A general linear model was used to evaluate the association between lung function and HRQOL scores after adjusting for potential confounders. Associations were quantified by using estimated changes and 95% confidence intervals (CIs) of HRQOL scores by the each unit increase of FVC or FEV_1_. We also estimated the changes (95% CI) in HRQOL scores for the second and third tertiles of lung function parameters, with comparison to the first tertile. Stratified analyses were conducted to investigate whether any of comorbidities modified the association between lung function and HRQOL. Comorbidities included gallstone, rhinitis, hypertension, hyperlipemia or diabetes.

To investigate the association between lung function and three facets of physical health domain, the participants were divided into low, middle and high group according to individual score for each facet. Generalized logistic model was used to estimate the odds ratio (OR) of each facet using high score group as the reference, while adjusting for the possible confounding effects of age, gender, BMI, marriage status, educational level, smoking status, passive smoking, alcohol consumption and physical activity. All analyses were performed using SAS version 9.4 (SAS Institute, Cary, North Carolina). *p* < 0.05 was considered statistically significant. Tests were two-tailed. Our study is reported according to the STROBE reporting guidelines.

## 3. Results

The characteristics of the 8398 participants (4837 men = 57.60%) are shown in Table 1. The mean age of the participants was 63.51 years. Participants with poor lung function were older and more likely men compared with those with normal lung function. And the poor lung function group had more current smokers, current drinkers and chronic disease patients such as hypertension and diabetes than the normal lung function group. No significant differences were found between the groups with respect to exercise and hyperlipemia.

The lung function indexes and the HRQOL scores of normal and poor lung function groups are shown in Table 2. All lung function indexes including those reflect small airway function in the normal lung function group were significantly higher than those in the group of poor lung function. The scores of the total HRQOL, physical health domain, psychological state domain and social relationships domain for the participants in normal lung function group were significantly higher than those in the group of poor lung function. No significant difference of environment domain score was observed between normal and poor lung function groups.

The association between FVC or FEV_1_ and HRQOL analyzed by the general linear model is shown in Table 3 and Table 4. In the continuous analysis, each unit increase in FVC was associated with a 1.36, 2.90, 1.27, 0.80, 0.49 score increase in the total HRQOL, physical health domain, psychological state domain, social relationships domain and environment domain scores, respectively (*p* < 0.05) (Table 3). After adjusting for all confounders, each unit increase in FVC was associated only with a 0.63, 1.67, 0.49 score increase in the total HRQOL, physical health domain and social relationships domain scores, respectively (*p* < 0.05). In the categorical analysis, significantly monotonic increase of the total and physical health domain scores was observed when FVC elevated after adjusting for all confounders. There was a similar association between FEV_1_ and HRQOL scores. And after adjusting all confounders, each unit increase in FEV_1_ was associated with a 0.53, 1.46 increase in the total HRQOL and physical health domain scores respectively, but no significant association was found between FEV_1_ level and psychological state domain, social relationships domain and environment domain scores in both continuous analysis and categorical analysis (*p* < 0.05).

To explore whether comorbidities (including gallstone, rhinitis, hypertension, hyperlipemia and diabetes) modified the association between lung function and HRQOL, we conducted stratified analyses in comorbidities participants and no comorbidities participants (Table 5). With adjustment for potential confounders, we found a significant positive association between FVC and HRQOL scores among no comorbidities participants. Each unit increase in FVC was associated with a 1.31, 1.76, 1.10, 1.40, 0.96 score increase in the total HRQOL, physical health domain, psychological state domain, social relationships domain and environment domain scores, respectively (*p* < 0.05). With regard to FEV_1_, it was associated with a 1.06, 1.64, 1.21 score increase in the total HRQOL, physical health domain and social relationships domain scores, respectively (*p* < 0.05). Among participants with comorbidities, positive association was only found between FVC and physical health domain and environment domain scores. And no association was observed between FEV_1_ and any HRQOL scores.

**Figure 1:**
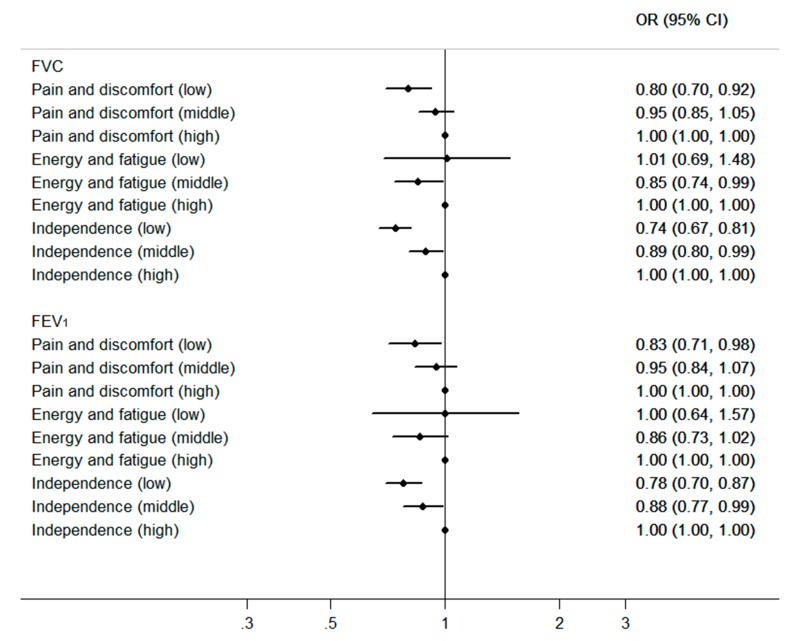
ORs (95%CI) for association between lung function (FVC and FEV_1_) and facet scores of physical health domain *. * The groups were defined as individuals with low, middle and high score, and the low score group was the reference. Adjusted for age, gender, BMI, marriage status, educational level, smoking status, passive smoking, alcohol consumption and physical activity.

Because the physical health domain played an important role in the relationship between lung function and HRQOL, we further analyzed the association between lung function and facet scores (pain and discomfort, energy and fatigue and independence) of physical health domain with adjustment for potential confounders (Figure 1). Compared with the high score group of pain and discomfort facet, we found that the odds ratio (OR) of FVC was 0.95 (95%CI, 0.85 to 1.05) in the middle score group and 0.80 (95%CI, 0.70 to 0.92) in the low score group. In the independence facet, the OR of FVC was 0.89 (95%CI, 0.80 to 0.99) in the middle score group and 0.74 (95%CI, 0.67 to 0.81) in the low score group, compared with the reference group. No significant positive association was found between FVC and energy and fatigue facet score. A similar relationship appeared between FEV_1_ and the facets.

## 4. Discussion

Our study provided evidence that lung function (FVC and FEV_1_) was positively associated with HRQOL scores in a large middle-aged and older Chinese population, which confirmed findings from previous studies [18,19]. After adjustment for potential confounders, the results suggested that FVC and FEV_1_ reduction was associated with decreased scores in the total HRQOL and physical health domain in the analysis which lung function parameters used as continuous or categorical variables. Further stratified analyses indicated similar association between FVC or FEV_1_ and HRQOL among the participants without any comorbidities. In present study, the association of FVC and psychological state domain and environment domain scores, and the association of FEV_1_ and psychological state domain, social relationships domain and environment domain scores, were not statistically significant when the relevant confounders were adjusted.

Possible reasons might be the fact that some demographic or physical characteristics such as age, gender, and BMI were related to HRQOL [20]. The relationship between lung function and physical health score in this study was in accordance with other studies [21,22]. However, a different report by Abbott and colleagues suggested that %pred FEV_1_ was significantly associated with all domains of HRQOL including physical health, psychological state, social relationships and so on in 234 cystic fibrosis patients [13]. A previous study also found 180 patients with COPD had a deterioration of psychosocial domain as well as a decline in physical health domain [23]. These studies reported more domains were associated with lung function in addition to physical health domain. Several reasons may explain the difference between those studies and ours. The first is that research participants in these studies were clinical patients with respiratory diseases such as COPD. Their lung function levels were far lower than the subjects in this study. Long-term illness can also affect other domains in HRQOL. The second is the number of participants, a small sample size may lead to unstable results. The third is the method used for HRQOL scores. The St. George’s Respiratory Questionnaire (SGRQ), the Clinical COPD Questionnaire (CCQ), the EuroQol-5D (EQ-5D), the Cystic Fibrosis Quality of Life Questionnaire (CFQOL) and the Medical Outcome Study Short-Form 36 Health Survey (SF-36) have all been used to evaluated HRQOL in previous studies. A Simon Pickard has proved that disease-specific instruments scores, such as the St. George’s Respiratory Questionnaire (SGRQ), were moderately correlated with FEV_1_, while generic instruments (i.e., EQ-5D and SF-36) scores had trivial levels of correlation with FEV_1_ [24]. We used the generic instrument, the World Health Organization Quality of Life-BREF to evaluate the aspect of health, daily life and well-being.

Published studies have demonstrated that comorbidities could decrease HRQOL of the patients with respiratory diseases [25,26,27]. To avoid modification by comorbidities, we employed stratified analyses. Our results suggested the association between FVC or FEV_1_ and the total HRQOL and physical health domain didn’t change among the participants without comorbidities. In participants with comorbidities, significant associations between lung function and the total HRQOL or most domains were not observed. Several kinds of comorbidities may plan different effects on HRQOL and cover up possible connections.

In this study, we further evaluated the relationship between the lung function and three facets scores of physical health domain. Increased OR among higher grades of pain and discomfort facet and independence facet indicated that the role of lung function on physical health score might be played through affecting pain and discomfort facet and independence facet. FVC and FEV_1_ in the low pain and discomfort facet group were 0.20 and 0.17 times lower than the reference group respectively. The reduction of the lung function reflected the severity of respiratory diseases and the symptoms of respiratory diseases might lead to physical discomfort or body pain. Shoup has found the dyspnea symptom to be the most significant factor related to HRQOL in adults with obstructive lung disease [28]. In the low independence facet group, FVC and FEV_1_ were 0.26 and 0.22 times lower than the high group respectively. It could indicate that the independence facet, which represents the capability for action, living or working, might decrease the most with the decline of lung function. A decline in FVC and FEV_1_ were due to increased airways resistance and decreased lung compliance [29]. These may result in activity limitation and decreased independence.

The strength of this study includes a large number of research participants, face to face interviews, and survey quality control. However, the limitations of this study should be acknowledged. Due to the cross-sectional study, we couldn’t determine the causality of the association between lung function and HRQOL. Besides, our participants were middle-aged and older people. A relatively higher percentage of COPD in elderly patients than younger ones may have an impact on HRQOL [23]. Another limitation was that we didn’t consider the effect of income. A cross-sectional study which included 2734 COPD patients found that higher income level had a higher HRQOL score [30].

## 5. Conclusions

In conclusion, we found that lung function was significantly associated with HRQOL, especially physical health domain and its facets, among middle-aged and elderly people in China. Our findings suggest that more attention should be paid to physical health domain for people with decreased lung function.

## Figures and Tables

**Table 1:** Baseline characteristics of Dongfeng-Tongji cohort participants according to normal lung function and poor lung function.

	Total	Lung Function		*p* Value
		Normal Lung Function †	Poor Lung Function ‡	
N	8398	5197	3201	
Sex (Male, N, %)	3561 (42.40)	1903 (36.62)	1658 (51.80)	<0.0001 *
Age (y, mean ± SD)	63.51 ± 8.06	62.11 ± 7.74	65.79 ± 8.07	<0.0001 *
BMI (kg/m^2^, mean ± SD)	24.08 ± 3.22	24.13 ± 3.10	24.00 ± 3.41	0.0591
Marriage (N, %)	7459 (88.82)	4676 (89.97)	2783 (86.94)	<0.0001 *
Education				<0.0001 *
<9y (N, %)	4820 (57.39)	2793 (53.740)	2027 (63.32)	
9–12y (N, %)	2532 (30.15)	1693 (32.58)	839 (26.21)	
>12y (N, %)	1046 (12.46)	711 (13.68)	335 (10.47)	
Smoking				<0.0001 *
No smoking (N, %)	6176 (73.54)	4070 (78.31)	2106 (65.79)	
Current smoking (N, %)	1331 (15.85)	677 (13.03)	654 (20.43)	
Quit smoking (N, %)	891 (10.61)	450 (8.66)	441 (13.78)	
Passive smoking (N, %)	2495 (29.71)	1629 (31.35)	866 (27.05)	<0.0001 *
Drinking				<0.0001 *
No drinking (N, %)	5846 (69.61)	3705 (71.29)	2141 (66.89)	
Current drinking (N, %)	2178 (25.93)	1312 (25.25)	866 (27.05)	
Quit drinking (N, %)	374 (4.45)	180 (3.46)	194 (6.06)	
Exercise (N, %)	7606 (90.57)	4718 (90.78)	2888 (90.22)	0.3926
Hypertension (N, %)	2771 (33.00)	1576 (30.33)	1195 (37.33)	<0.0001 *
Hyperlipemia (N, %)	1549 (18.44)	961 (18.49)	588 (18.37)	0.8885
Diabetes (N, %)	943 (11.23)	530 (10.20)	413 (12.90)	0.0001 *

* *p* < 0.05 is considered statistically significant. † Normal lung function: FVC% ≥ 80% and FEV_1_/FVC ≥ 70%. ‡ Poor lung function: FVC% < 80% and FEV_1_/FVC ≥ 70% or FEV_1_/FVC < 70%.

**Table 2:** Lung function indexes and HRQOL scores of Dongfeng-Tongji cohort participants according to normal lung function and poor lung function.

	Total	Lung Function		*p* Value
		Normal Lung Function †	Poor Lung Function ‡	
N	8398	5197	3201	
FVC (L)	2.44 ± 0.70	2.75 ± 0.58	1.93 ± 0.56	<0.0001 *
FEV_1_ (L)	2.10 ± 0.60	2.37 ± 0.49	1.66 ± 0.48	<0.0001 *
FEV_1_/FVC (%)	86.45 ± 10.79	86.64 ± 7.95	86.13 ± 14.23	<0.0001 *
MMF (L/s)	2.64 ± 1.01	2.87 ± 0.85	2.27 ± 1.13	<0.0001 *
PEF (L/s)	4.38 ± 1.90	4.81 ± 1.85	3.69 ± 1.78	<0.0001 *
FEF25 (L/s)	3.44 ± 1.98	3.68 ± 2.04	3.04 ± 1.82	<0.0001 *
FEF50 (L/s)	2.97 ± 1.25	3.28 ± 1.09	2.47 ± 1.33	<0.0001 *
FEF75 (L/s)	2.02 ± 1.49	2.30 ± 1.62	1.57 ± 1.11	<0.0001 *
Total scores	75.40 ± 10.51	75.88 ± 10.44	74.63 ± 10.57	<0.0001 *
Physical health domain score	75.37 ± 17.05	76.35 ± 16.68	73.78 ± 17.52	<0.0001 *
Psychological state domain score	73.58 ± 13.07	73.89 ± 13.15	73.08 ± 12.92	<0.0001 *
Social relationships domain score	74.50 ± 12.75	74.95 ± 12.70	73.77 ± 12.79	<0.0001 *
Environment domain score	78.15 ± 12.27	78.32 ± 12.35	77.88 ± 12.14	0.8377

* *p* < 0.05 is considered statistically significant. † Normal lung function: FVC% ≥ 80% and FEV_1_/FVC ≥ 70%. ‡ Poor lung function: FVC% < 80% and FEV_1_/FVC ≥ 70% or FEV_1_/FVC < 70%.

**Table 3:** Association between FVC and HRQOL scores in Dongfeng-Tongji cohort participants.

		Estimated Changes (95% CI) by Continuous FVC		Estimated Changes (95% CI) by Tertile of FVC			
		β	*p* Value	T1 (<2.12 L)	T2 (2.12–2.69 L)	T3 (≥2.69 L)	*p* Trend
N				2767	2807	2824	
Median (L)				1.78	2.39	3.08	
Total score	Univariate model	1.36 (1.04,1.69)	<0.0001 *	0 (reference)	1.43 (0.88,1.98) *	2.12 (1.57,2.67) *	<0.0001 *
	Model 1 †	0.83 (0.46,1.20)	<0.0001 *	0 (reference)	0.73 (0.17,1.30) *	1.18 (0.56,1.80) *	0.0002 *
	Model 2 ‡	0.84 (0.47,1.21)	<0.0001 *	0 (reference)	0.75 (0.19,1.32) *	1.20 (0.58,1.82) *	0.0002 *
	Model 3 §	0.63 (0.26,1.00)	0.0010 *	0 (reference)	0.61 (0.04,1.17) *	0.89 (0.27,1.51) *	0.0056 *
Physical health domain score	Univariate model	2.90 (2.38,3.42)	<0.0001 *	0 (reference)	2.33 (1.44,3.22) *	4.27 (3.38,5.16) *	<0.0001 *
	Model 1 †	1.85 (1.25,2.45)	<0.0001 *	0 (reference)	0.98 (0.07,1.89) *	2.36 (1.36,3.36) *	<0.0001 *
	Model 2 ‡	1.88 (1.29,2.48)	<0.0001 *	0 (reference)	1.03 (0.11,1.94) *	2.41 (1.41,3.41) *	<0.0001 *
	Model 3 §	1.67 (1.07,2.28)	<0.0001 *	0 (reference)	0.89 (−0.02,1.80)	2.08 (1.07,3.08) *	<0.0001 *
Psychological state domain score	Univariate model	1.27 (0.87,1.67)	<0.0001 *	0 (reference)	1.58 (0.90,2.27) *	2.12 (1.44,2.81) *	<0.0001 *
	Model 1 †	0.50 (0.04,0.96)	0.0343 *	0 (reference)	0.82 (0.11,1.52) *	0.88 (0.10,1.65) *	0.0294 *
	Model 2 ‡	0.51 (0.05,0.97)	0.0309 *	0 (reference)	0.83 (0.13,1.54) *	0.89 (0.12,1.67) *	0.0266 *
	Model 3 §	0.25 (−0.21,0.72)	0.2850	0 (reference)	0.64 (−0.06,1.35)	0.54 (−0.24,1.32)	0.1864
Social relationships domain score	Univariate model	0.80 (0.41,1.19)	<0.0001 *	0 (reference)	1.09 (0.42,1.76) *	1.24 (0.58,1.91) *	0.0003 *
	Model 1 †	0.71 (0.26,1.16)	0.0021 *	0 (reference)	0.77 (0.08,1.46) *	1.05 (0.29,1.80) *	0.0074 *
	Model 2 ‡	0.72 (0.27,1.17)	0.0018 *	0 (reference)	0.78 (0.09,1.47) *	1.06 (0.30,1.82) *	0.0066 *
	Model 3 §	0.49 (0.03,0.95)	0.0356 *	0 (reference)	0.62 (−0.08,1.31)	0.72 (−0.04,1.49)	0.0670
Environment domain score	Univariate model	0.49 (0.12,0.87)	0.0103 *	0 (reference)	0.73 (0.08,1.37) *	0.84 (0.19,1.48) *	0.0214 *
	Model 1 †	0.25 (−0.19,0.69)	0.2601	0 (reference)	0.37 (−0.29,1.04)	0.44 (−0.29,1.17)	0.2444
	Model 2 ‡	0.26 (−0.18,0.69)	0.2469	0 (reference)	0.38 (−0.28,1.05)	0.45 (−0.28,1.18)	0.2324
	Model 3 §	0.09 (−0.35,0.54)	0.6739	0 (reference)	0.27 (−0.40,0.94)	0.22 (−0.52,0.95)	0.5796

* *p* < 0.05 is considered statistically significant. † Model 1: adjusted for age and gender. ‡ Model 2: adjusted for variables in model 1 plus BMI. § Model 3: adjusted for variables in model 2 plus marriage status, educational level, smoking status, passive smoking, alcohol consumption and physical activity.

**Table 4:** Association between FEV_1_ and HRQOL scores in Dongfeng-Tongji cohort participants.

		Estimated Changes (95% CI) by Continuous FEV_1_		Estimated Changes (95% CI) by Tertile of FEV_1_			*p* Trend
		β	*p* Value	T1 (<1.86 L)	T2 (1.86–2.33 L)	T3 (≥2.33 L)	
N				2797	2769	2832	
Median (L)				1.55	2.09	2.64	
Total score	Univariate model	1.54 (1.17,1.92)	<0.0001 *	0 (reference)	1.53 (0.98,2.08) *	2.09 (1.54,2.64) *	<0.0001 *
	Model 1 †	0.73 (0.29,1.16)	0.0011 *	0 (reference)	0.76 (0.19,1.33) *	0.93 (0.31,1.56) *	0.0031 *
	Model 2 ‡	0.76 (0.33,1.20)	0.0006 *	0 (reference)	0.78 (0.22,1.35) *	0.97 (0.35,1.60) *	0.0021 *
	Model 3 §	0.53 (0.09,0.97)	0.0175 *	0 (reference)	0.61 (0.04,1.17) *	0.69 (0.07,1.32) *	0.0287 *
Physical health domain score	Univariate model	3.21 (2.60,3.82)	<0.0001 *	0 (reference)	3.01 (2.12,3.90) *	3.88 (2.99,4.77) *	<0.0001 *
	Model 1 †	1.62 (0.92,2.33)	<0.0001 *	0 (reference)	1.51 (0.59,2.42) *	1.47 (0.46,2.47) *	0.0040 *
	Model 2 ‡	1.71 (1.00,2.41)	<0.0001 *	0 (reference)	1.56 (0.65,2.47) *	1.55 (0.55,2.56) *	0.0022 *
	Model 3 §	1.46 (0.75,2.17)	<0.0001 *	0 (reference)	1.38 (0.47,2.30) *	1.25 (0.24,2.27) *	0.0142 *
Psychological state domain score	Univariate model	1.50 (1.03,1.97)	<0.0001 *	0 (reference)	1.43 (0.74,2.11) *	2.19 (1.51,2.87) *	<0.0001 *
	Model 1 †	0.47 (−0.07,1.02)	0.0890	0 (reference)	0.64 (−0.06,1.35)	0.80 (0.02,1.58) *	0.0421 *
	Model 2 ‡	0.50 (−0.05,1.04)	0.0732	0 (reference)	0.66 (−0.05,1.37)	0.83 (0.05,1.61) *	0.0357 *
	Model 3 §	0.22 (−0.33,0.77)	0.4258	0 (reference)	0.45 (−0.25,1.16)	0.51 (−0.27,1.16)	0.1979
Social relationships domain score	Univariate model	0.92 (0.46,1.38)	<0.0001 *	0 (reference)	1.03 (0.36,1.69) *	1.31 (0.65,1.98) *	0.0001 *
	Model 1 †	0.68 (0.14,1.21)	0.0130 *	0 (reference)	0.62 (−0.07,1.31)	0.98 (0.22,1.74) *	0.0116 *
	Model 2 ‡	0.70 (0.17,1.23)	0.0100 *	0 (reference)	0.64 (−0.06,1.33)	1.00 (0.24,1.76) *	0.0096 **
	Model 3 §	0.46 (−0.07,1.00)	0.0914	0 (reference)	0.45 (−0.25,1.14)	0.71 (−0.06,1.47)	0.0692
Environment domain score	Univariate model	0.54 (0.10,0.98)	0.0169 *	0 (reference)	0.66 (0.02,1.31) *	0.97 (0.33,1.61) *	0.0031 *
	Model 1 †	0.13 (−0.38,0.64)	0.6221	0 (reference)	0.27 (−0.40,0.93)	0.49 (−0.25,1.22)	0.1925
	Model 2 ‡	0.15 (−0.37,0.66)	0.5742	0 (reference)	0.28 (−0.39,0.94)	0.51 (−0.23,1.24)	0.1755
	Model 3 §	−0.02 (−0.54,0.50)	0.9439	0 (reference)	0.15 (−0.52,0.82)	0.30 (−0.44,1.04)	0.4218

* *p* < 0.05 is considered statistically significant. † Model 1: adjusted for age and gender. ‡ Model 2: adjusted for variables in model 1 plus BMI. § Model 3: adjusted for variables in model 2 plus marriage status, educational level, smoking status, passive smoking, alcohol consumption and physical activity.

**Table 5:** Association between lung function (FVC and FEV_1_) and HRQOL scores, stratified by comorbidities and no-comorbidities.

		Estimated Changes (95% CI) by Continuous FVC ‡		Estimated Changes (95% CI) by Continuous FEV_1_ ‡	
		β	*p* Value	β	*p* Value
No comorbidities † (*n* = 3871)	Total score	1.31 (0.76,1.85)	<0.0001 *	1.06 (0.42,1.69)	0.0011 *
	Physical health domain score	1.76 (0.94,2.59)	<0.0001 *	1.64 (0.67,2.60)	0.0009 *
	Psychological state domain score	1.10 (0.42,1.78)	0.0015 *	0.76 (−0.04,1.56)	0.0626
	Social relationships domain score	1.40 (0.73,2.06)	<0.0001 *	1.21 (0.43,2.00)	0.0024 *
	Environment domain score	0.96 (0.32,1.60)	0.0031 *	0.61 (−0.13,1.36)	0.1077
Comorbidities † (*n* = 4527)	Total score	−0.04 (−0.55,0.47)	0.8781	−0.04 (−0.64,0.57)	0.9055
	Physical health domain score	1.32 (0.48,2.16)	0.0022 *	0.97 (−0.02,1.97)	0.0557
	Psychological state domain score	−0.49 (−1.14,0.15)	0.1303	−0.27 (−1.03,0.49)	0.4821
	Social relationships domain score	−0.31 (−0.94,0.32)	0.3346	−0.22 (−0.97,0.52)	0.5579
	Environment domain score	−0.68 (−1.29,−0.06)	0.0302 *	−0.63 (−1.35,0.10)	0.0889

* *p* < 0.05 is considered statistically significant. † Comorbidities: including gallstone, rhinitis, hypertension, hyperlipemia and diabetes. ‡ Adjusted for age, gender, BMI, marriage status, educational level, smoking status, passive smoking, alcohol consumption and physical activity.
